# Effects of multigrain rice and white rice on periodontitis: an analysis using data from the Korea National Health and Nutrition Examination Survey 2012-2015

**DOI:** 10.4178/epih.e2023063

**Published:** 2023-07-03

**Authors:** Seung-Hee Ryu, Zi-lan Wang, Seon-Jip Kim, Hyun-Jae Cho

**Affiliations:** Department of Preventive Dentistry & Public Oral Health, School of Dentistry and Dental Research Institute, Seoul National University, Seoul, Korea

**Keywords:** Periodontitis, Whole grains, Diet, Diabetes mellitus, Glycemic control

## Abstract

**OBJECTIVES:**

Numerous studies have investigated the efficacy of whole grains; however, research on multigrain remains limited. Grains exhibit combined positive effects against various diseases. The purpose of this study was to examine the impact of multigrain and white rice consumption on periodontitis.

**METHODS:**

We analyzed data from the Korea National Health and Nutrition Examination Survey V-3 and VI, collected between 2012 and 2015, which included 12,450 patients (4,859 male and 7,591 female) aged 19-64 years. The World Health Organization’s Community Periodontal Index (CPI) was utilized to assess the presence of periodontitis, with periodontitis defined as a CPI index score of ≥3. Multivariable logistic regression analysis was performed after adjusting for potential confounding variables.

**RESULTS:**

The group that consumed only multigrain rice was less likely to have periodontitis than the group that consumed only white rice (odds ratio [OR], 0.80; 95% confidence interval [CI], 0.69 to 0.93). When stratified by sex, the risk of periodontitis demonstrated a 24% decrease in female who consumed only multigrain rice (OR, 0.76; 95% CI, 0.62 to 0.93). A similar result was observed in the age group of 40-64 years (OR, 0.84; 95% CI, 0.71 to 0.99). In the diabetes stratification model, the normal group that consumed only multigrain rice exhibited a 25% decrease in the odds of periodontitis (OR, 0.75; 95% CI, 0.62 to 0.91).

**CONCLUSIONS:**

Our findings suggest that the prevalence of periodontitis may vary depending on the type of rice consumed.

## GRAPHICAL ABSTRACT

[Fig f2-epih-45-e2023063]

## INTRODUCTION

Rice is a staple food in the Korean diet [1]. The phrase “Have you eaten rice yet?” is a common greeting among Koreans [2]. In 2021, the annual per capita rice consumption in Korea was 56.9 kg [3], making Korea the 15th largest consumer of rice worldwide. This is quite significant, given the consumption relative to the population [4]. Rice is consumed in various forms, such as white, brown, black, and multigrain rice. Whole grains, including multigrain rice, contain dietary fiber and numerous protective components, such as resistant starch, trace minerals, phenolic compounds that function as antioxidants, and phytoestrogens with potential hormonal effects [5].

Several studies have revealed the benefits of a whole-grain diet [6-8]. Incorporating whole grains or a high-fiber diet is crucial in diabetes management, as it enhances glycemic control, blood lipid levels, body weight, and inflammation, while also reducing the risk of early mortality [9]. Consuming whole grains may decrease the likelihood of periodontitis due to its impact on inflammation and glycemic control [10]. Periodontitis is a chronic inflammatory condition that gradually leads to the deterioration of the tissues supporting the teeth [11]. This prevalent and severe disease is found globally and is expected to increase alongside the growing aging population [12]. A low intake of grains has been associated with periodontal diseases, and individuals who consume limited amounts of whole grains are more prone to developing severe periodontitis [13]. The protective effect of whole grains on the progression of periodontal disease can aid in managing glucose intolerance and reducing the risk of insulin resistance [14,15].

Whole grains are nutritionally superior to refined grains [16]. During the refining process, white rice, a refined grain, loses numerous protective components [17]. The primary sources of whole grains are ready-to-eat, cooked, and processed grains, such as cereals and bread [18]. Multigrain rice, a type of cooked grain, shares characteristics with whole grains [19] and is more effective in preventing diseases compared to refined grains [20,21].

Numerous studies have examined the impact of whole grains on various diseases [5,10,22], but none have explored the effect of multigrain rice on periodontitis. Consequently, we aimed to investigate the association between the prevalence of periodontitis and the type of rice consumed. We hypothesized that the intake of multigrain rice may help prevent periodontitis due to its influence on complex factors, such as glycemic control, akin to the effects observed from whole grain consumption.

## MATERIALS AND METHODS

### Data source and study population

**Figure 1.**
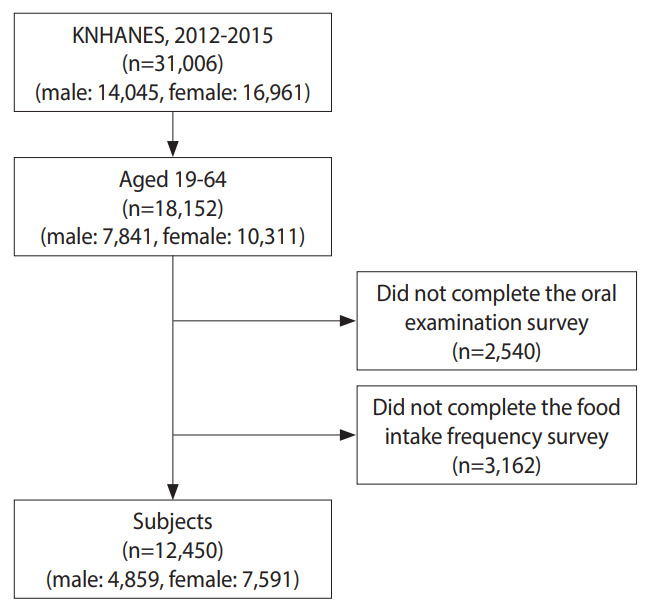
Flow chart of sample selection. KNHANES, Korea National Health and Nutrition Examination Surveys.

In this study, we utilized data from Korea National Health and Nutrition Examination Surveys (KNHANES) V-3 (2012) and VI (2013-2015). The KNHANES is a cross-sectional survey that assesses the overall health and nutritional status of a representative Korean population, conducted by the Korea Disease Control and Prevention Agency (KDCA). The survey’s sampling protocol involves a multistage probability group, stratifying representative samples of non-institutional civilians in Korea. This study utilized individual data from 18,152 Koreans aged 19-64 years who participated in the KNHANES from 2012 to 2015. Participants who did not complete the oral examination (n=2,540) or food intake frequency survey (n=3,162) were excluded. Consequently, we enrolled 12,450 individuals in the study, including 4,859 male and 7,591 female (Figure 1). Data on socio-demographic characteristics, oral health-related variables, general health status indicators, and consumption frequencies of white and multigrain rice were collected from the KNHANES (2012-2015).

### The proportion of multigrain to white rice

In KNHANES V and VI, “multigrain rice” was defined as rice composed of brown rice, barley, beans, and red beans. The food intake frequency in KNHANES was assessed by asking participants the following question: “What is your average intake frequency over the past year?” Participants’ responses were categorized into 9 groups: rarely, once a month, 2-3 times a month, once a week, 2-4 times a week, 5-6 times a week, once a day, twice a day, and three times a day. Only those who consumed multigrain or white rice at least twice per day were included, while those who consumed other foods and not rice daily were excluded. Participants were then divided into different groups based on the ratio of multigrain and white rice they consumed. The groups are as follows: 100% multigrain rice; multigrain rice ≤ 50% and white rice > 50%; multigrain rice > 50% and white rice ≤ 50%; and 100% white rice.

### Socio-demographic and health behavior variables

The following socio-demographic characteristics were identified as confounders: sex, age, household income, and education level. Participants were divided into 4 groups based on household income. Additionally, 4 groups were established based on education level according to the Korean education level classification code, which includes: below elementary school graduation, middle school, high school, and above university graduation. Oral health-related variables encompassed tooth brushing frequency, interdental brush use, dental floss use, and Community Periodontal Index (CPI). Tooth brushing frequency was categorized as either less than or at least twice a day. Interdental brush and dental floss use were classified as “yes” or “no” depending on their usage. General health status indicators included smoking habits, diabetes mellitus, hypercholesterolemia, hypertension, and body mass index (BMI). Participants were classified as current smokers, ex-smokers, and non-smokers based on their smoking habits. Regarding the presence or absence of diabetes, participants were classified as normal (fasting glucose level < 100 mg/dL), impaired fasting glucose (fasting glucose level ≥ 100 and ≤ 125 mg/dL), and diabetes (fasting glucose level ≥ 126 mg/dL, use of antidiabetics, administration of insulin injections, or diagnosis by a physician). Hypercholesterolemia was defined as a total cholesterol level ≥ 240 mg/dL or use of cholesterol medication. Hypertension was classified as normal (systolic blood pressure [SBP]/diastolic blood pressure [DBP] < 120/80 mmHg); prehypertension (SBP ≥ 120 and < 140 mmHg and DBP ≥ 80 and < 90 mmHg); and hypertension (SBP/DBP ≥ 140/90 mmHg or use of antihypertensive drugs). BMI was classified as normal (BMI ≥ 18.5 and < 25.0 kg/m^2^), low (BMI < 18.5 kg/m^2^), and high (BMI ≥ 25.0 kg/m^2^).

### Periodontal index

The CPI, initially developed by the World Health Organization, is utilized to evaluate periodontal status [23]. It serves as an indicator of the need for periodontal treatment among residents or specific groups within a community. Periodontitis is defined as a CPI score of ≥ 3. The KDCA carries out investigations through public health dentists who have received training and field quality management education to conduct standardized assessments. This training takes place over three days, consisting of theoretical education, photography education, and a mock examination. Notably, periodontal tissue tests are conducted twice daily for four days, with a total of ≥ 8 repetitions, to ensure proper pressure is maintained during periodontal probing. Between 2012 and 2015, the CPI’s kappa index ranged from 0.692 to 0.799 in the KNHANES [24].

### Statistical analysis

All research data were analyzed using SPSS version 26.0 (IBM Corp., Armonk, NY, USA). Complex sample survey data were employed to account for multilevel, stratified, unequal selection probabilities, or clustered sample designs related to KNHANES (2012-2015). Appropriate sample weights were applied for each data collection. Multivariable logistic regression analysis was utilized to calculate the association between consumption of white or multigrain rice and periodontitis, as determined by adjusted odds ratios (ORs) and 95% confidence intervals (CIs). The logistic regression analysis was adjusted for potential confounders. Chi-square tests were employed to compare the prevalence of periodontitis among patients with and without diabetes. Statistical significance was set at a p-value of < 0.05.

### Ethics statement

The KDCA Institutional Review Board (IRB) approved the survey protocol and secondary data use (IRB approval: #2012-01EXP-01-2C, 2013-07CON-03-4C, and 2013-12EXP-03-5C). For KNHANES VI-3 (2015), the survey was conducted without deliberation according to the IRB of the KDCA.

## RESULTS

Table 1 presents the characteristics of participants grouped by the presence or absence of periodontitis. Out of the 22,601 participants, 6,189 (27.4%) had periodontitis, while 16,412 (72.6%) did not. Regardless of periodontitis status, those who consumed only multigrain rice were the most prevalent in both groups, with 52.0% in the periodontitis group and 51.2% in the non-periodontitis group. Participants who consumed only white rice accounted for 28.8% and 28.0% of the population in the periodontitis and nonperiodontitis groups, respectively. The distribution of rice consumption showed similar patterns, regardless of periodontitis status (Table 1).

The results of logistic regression analysis for the effect of rice dietary patterns on periodontitis prevalence are shown in Table 2. In model 3, after adjusting for all variables, participants who consumed only multigrain rice were 20% less likely to have periodontitis than those who consumed only white rice (OR, 0.80; 95% CI, 0.69 to 0.93). Additionally, a trend analysis was conducted to identify trends between rice intake patterns and periodontitis prevalence. In all models, since the p-value for trend was < 0.05, the prevalence of periodontitis according to rice intake patterns showed a consistent direction (Table 2).

Table 3 presents the results of the logistic regression analysis stratified by sex, age, and diabetes. Female who consumed only multigrain rice were 24% less likely to have periodontitis than those who consumed only white rice (OR, 0.76; 95% CI, 0.62 to 0.93). Similarly, this result was found in the age group of 40 years or older. The age group of 40-64 years that consumed only multigrain rice showed a 16% reduced risk of periodontitis (OR, 0.84; 95% CI, 0.71 to 0.99). In the stratification model classified by diabetes status, a similar decrease was observed only in the normal group. In the normal group, participants who consumed only multigrain rice were 25% less likely to have periodontitis than those who consumed only white rice (OR, 0.75; 95% CI, 0.62 to 0.91) (Table 3).

The distribution of sex, age, and diabetes according to rice dietary patterns is displayed in Table 4. When classified by sex, 39.2% of male and 64.1% of female were in the multigrain rice consumption group. By age, 36.8% of the 19-39-year-old group consumed only white rice, higher than the 21.8% observed in the 40-64-year-old group. Conversely, 58.1% of the 40-64-year-old group consumed only multigrain rice, higher than the 43.9% observed in the 19-39-year-old group. When classified by diabetes status, 60.8% of patients with diabetes consumed only multigrain rice, compared to the normal group. In the normal group, 51.8% consumed only multigrain rice, and 29.3% consumed only white rice, higher than the 18.3% observed in the diabetes group (Table 4).

Table 5 shows the average blood glucose level stratified by rice intake type according to the presence of diabetes and periodontitis. The diabetic group exhibited a difference in blood glucose levels between participants who consumed only white rice and those who consumed both white and multigrain rice among patients with periodontitis. The blood glucose level did not differ among stratified groups (Table 5).

## DISCUSSION

In this study, we investigated the effects of dietary patterns involving white and multigrain rice on periodontitis in Koreans aged 19-64 years. While previous studies have explored the relationship between whole grains and periodontitis, this is the first study to examine the impact of rice type consumption on periodontitis. We discovered that the risk of periodontitis decreased in the group that consumed only multigrain rice compared to the group that consumed only white rice. This difference in periodontitis prevalence based on the type of rice consumed could be attributed to the rice refining process. White rice is considered a refined grain with a high content of starch, which is a carbohydrate polymer that remains after the bran and germ of the whole grain are removed [19]. The refining process eliminates most of the naturally occurring vitamins, minerals, dietary fiber, lignans, phytoestrogens, phenolic compounds, and phytic acid [25]. As a result, unrefined whole grains are more nutritious than refined white rice [5].

To understand the mechanism, the effect of whole grains on glycemic control was observed. High fasting blood glucose levels are associated with C-reactive protein (CRP) levels and the risk of inflammation [26]. An elevation in CRP is linked to insulin resistance [27]. Tumor necrosis factor-α and interleukin (IL)-6 are derivatives of acute-phase proteins, including CRP, and both potentially contribute to insulin resistance by affecting intracellular insulin signaling [28]. Compared to healthy controls, patients with periodontitis exhibit elevated serum IL-6 and CRP levels [29]. Inflammatory disorders in hyperglycemia lead to both microvascular and macrovascular complications [30]. Complications arising from hyperglycemia cause damage to capillary function, decreased blood flow to tissues and organs, oxidative stress, elevated inflammatory processes through cytokine exposure, and severe periodontal disease [31]. Hyperglycemia is a major determinant of the risk, severity, and extent of periodontitis [32]. However, whole grains can attenuate the blood glucose response after a meal [33]. Since whole-grain starch is more resistant to digestion compared to refined starch [34], whole grains slow the digestion and absorption of carbohydrates in the intestine [35]. Dietary fiber also promotes satiety and increases digestion time [36], thus lowering blood glucose and insulin levels [37]. Therefore, the consumption of multigrain rice may be closely associated with a decreased risk of periodontitis. Although this study showed no significant difference in blood glucose levels between groups (Table 5), it was a cross-sectional study and only represented fasting glycemic results at the time of the examination. In reality, elevated postprandial glycemia may vary between groups.

Moreover, studies have examined the direct impact of whole grains on periodontitis. One study, which involved 34,160 male health professionals aged 45-75 years, suggested that consuming more than four servings of whole grains per day (1 serving=3/4 cup whole-grain cereal or 1 slice whole-wheat bread) could lower the risk of periodontitis [10]. However, some research has argued that whole grains do not have any significant effect on periodontal disease. A randomized controlled trial investigated the effects of tailored dietary interventions, such as fruit, vegetable, and wholegrain consumption, on chronic periodontitis in 51 hospital participants aged 30-65 years. The results demonstrated that wholegrain intake significantly increased the total antioxidant capacity in the intervention group six months after the intervention; however, there was no significant difference in the periodontal index [38]. The study attributed the lack of significant changes in the periodontal index to the possibility that the extent of dietary changes may not be enough to affect these indices, and that the number of participants and the duration of the dietary intervention may not be sufficient to produce significant differences.

The effect of the type of rice consumed on periodontitis was also examined in relation to sex, age, and the presence of diabetes. Upon hierarchical analysis, the risk of periodontitis was found to be lower in the group that consumed only multigrain rice, among female, in the age group of 40 years to 64 years, and in the normal fasting glucose level group (Table 3). In Korea, the age of 40 is considered a “life transition period,” a time when bodily changes necessitate management [39]. Given that dietary strategies change with age due to health concerns [40], the reduced odds could be attributed to the health behavior of consuming only multigrain rice. The 40-64-year-old group had a higher proportion of participants who consumed only multigrain rice compared to the 19-39-year-old group (Table 4). This was also true for female, as the proportion of those who consumed only multigrain rice was higher among female than male (Table 4). The lower risk of periodontitis in the multigrain rice-consuming group among female was thought to be due to the presence of phytoestrogens in whole grains. A high intake of phytoestrogens, which are phenolic compounds, has significant estrogenic/antiestrogenic effects in both animals and humans [41]. Epidemiological, laboratory, and clinical evidence suggest that phytoestrogens have a positive impact on bone mineral density [42,43]. There is a negative correlation between periodontal disease and bone density in postmenopausal female [44,45], and consuming multigrain rice may help alleviate this issue. However, while phytoestrogens seem to improve bone density, there is not enough research on their long-term effects [46].

When categorized by diabetes status, the risk of periodontitis decreased only in the normal group (Table 3). However, blood glucose levels significantly varied depending on the type of rice consumed in the normal group (Table 5). Thus, in the normal group, multigrain rice was estimated to have a more substantial direct effect on periodontitis compared to the effect of glycemic control. The proportion of participants consuming only multigrain rice was higher in the diabetic group than in the normal group (Table 4). For patients with diabetes, the high proportion of participants consuming only multigrain rice may be due to their adherence to doctor-recommended health behaviors, such as consuming multigrain rice. Furthermore, the American Diabetes Association emphasized the intake of fiber and whole grains as positive health behaviors to improve diabetes management in 2023 [47]. In diabetics, the health behavior of consuming multigrain rice may have a positive effect on blood glucose reduction (Table 5). Regardless of the presence or absence of periodontitis, the group that consumed only multigrain rice had lower average blood glucose levels than the group that consumed only white rice (Table 5). However, multigrain rice intake did not affect the relationship between diabetes and periodontitis (Table 3).

The association between diabetes and periodontal disease influenced the results. Diabetes has a bidirectional relationship with periodontal disease. According to a study that analyzed the relationship between the two diseases from an epidemiological perspective, patients with diabetes tended to have a higher prevalence, greater severity, and faster progression of periodontal disease than controls. Treating periodontal infections can help with blood glucose management and minimize the burden of diabetes complications [48]. A study that used large sample data to investigate the association between type 2 diabetes mellitus and periodontal disease in 4,343 United States adults aged 45 years reported a significantly higher prevalence of severe periodontitis in those with diabetes than in those without [49]. Another similar study confirmed that periodontal treatment affects metabolic regulation and reduces systemic inflammation in type 2 diabetes mellitus. Moreover, periodontal therapy is a necessary component in the development of treatment approaches that can reduce complications of diabetes [50].

The strength of this study lies in its large-scale analysis confirming the relationship between periodontitis and the consumption of multigrain and white rice. To minimize bias caused by the consumption of foods other than rice as a staple, we analyzed participants who ate rice more than twice a day, considering rice as their primary food source. We calculated statistical results by adjusting for oral health-related variables that act as significant confounding factors in periodontal disease in the final model of all tables. Furthermore, we computed the average value to investigate the effect of blood glucose levels, which are related to periodontitis, on dietary patterns. The blood glucose levels of diabetics were lower in the group that consumed only multigrain rice, and the risk of periodontitis decreased in the normal group without changes in blood glucose levels. However, this study has some limitations. First, since the food intake survey responses were based on self-reported data, there may have been recall bias in the relationship between periodontitis and the consumption of multigrain and white rice. Second, multicollinearity may exist because multigrain and white rice have alternative relationships. Therefore, in this study, we combined two highly correlated variables to create a new variable for analysis, and a trend analysis revealed that the step-by-step model produced results with the same directionality.

In this study, we examined the impact of different types of rice consumption on the risk of periodontitis using large-scale data from Korea. Despite the limitations of the cross-sectional design, our findings indicate that consuming multigrain rice at least twice daily is more effective in preventing periodontitis than eating white rice. These results suggest a greater preventive effect in healthy individuals compared to those with diabetes. However, due to insufficient data to establish a causal relationship, further prospective studies are necessary.

## Figures and Tables

**Figure f2-epih-45-e2023063:**
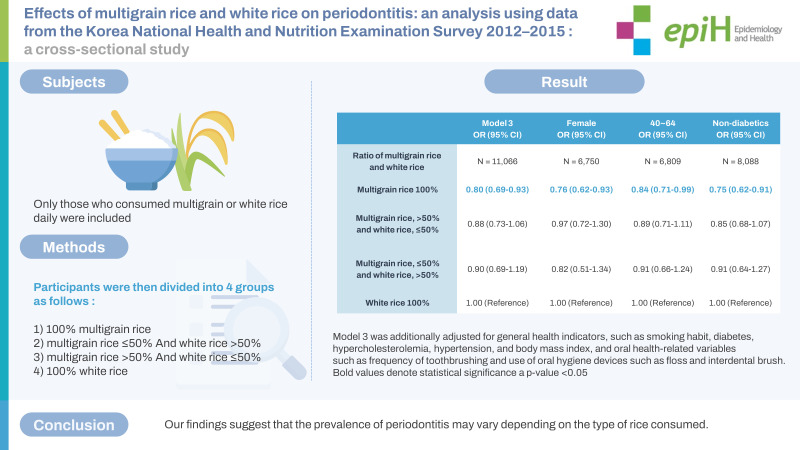


**Table 1.** Characteristics of participants according to the presence/absence of periodontitis (n=22,601)

Characteristics		Presence of periodontitis		p-value^1^
		No (n=16,412)	Yes (n=6,189)	
Age, mean±standard deviation		42.85±18.99	58.20±13.17	<0.001^2^
Sex				<0.001
	Male	6,597 (47.1)	3,206 (58.4)	
	Female	9,815 (52.9)	2,983 (41.6)	
Household income				<0.001
	Low	2,414 (11.9)	1,595 (21.2)	
	Middle low	4,031 (24.8)	1,668 (27.2)	
	Middle high	4,718 (30.5)	1,494 (26.9)	
	High	5,120 (32.8)	1,372 (24.8)	
Education level				<0.001
	≤Elementary school	3,569 (16.6)	1,982 (27.0)	
	Middle school	1,978 (12.3)	872 (14.4)	
	High school	4,959 (36.2)	1,696 (34.5)	
	≥University	4,964 (34.9)	1,168 (24.1)	
Tooth brushing frequency				<0.001
	Less than twice a day	1,450 (8.7)	949 (15.5)	
	More than twice a day	14,247 (91.3)	4,884 (84.5)	
Interdental brushing				0.041
	No	12,572 (79.8)	4,872 (81.7)	
	Yes	3,190 (20.2)	994 (18.3)	
Flossing				<0.001
	No	12,135 (76.0)	5,159 (86.8)	
	Yes	3,627 (24.0)	707 (13.2)	
Smoking				<0.001
	Current smoker	2,215 (17.5)	1,424 (29.3)	
	Ex-smoker	2,446 (16.3)	1,432 (25.3)	
	Non-smoker	11,057 (66.2)	2,980 (45.4)	
Diabetes mellitus				<0.001
	Normal	9,044 (75.8)	2,874 (56.3)	
	Impaired fasting glucose	2,413 (18.2)	1,451 (27.7)	
	Diabetes	976 (6.0)	919 (16.0)	
Hypercholesterolemia				<0.001
	Normal	10,604 (87.7)	4,165 (81.8)	
	Abnormal	1,827 (12.3)	1,077 (18.2)	
Hypertension				<0.001
	Normal	6,943 (56.1)	1,881 (36.6)	
	Prehypertension	3,123 (24.3)	1,349 (24.6)	
	Hypertension	3,332 (19.6)	2,521 (38.8)	
Body mass index				<0.001
	Low	1,227 (7.4)	158 (2.6)	
	Normal	10,568 (64.3)	3,594 (57.5)	
	High	4,585 (28.2)	2,433 (39.9)	
Ratio of multigrain rice and white rice				
	Multigrain rice 100%	5,331 (52.0)	1,751 (51.2)	0.007
	Multigrain rice, >50% and white rice, ≤50%	1,315 (14.9)	428 (15.2)	
	Multigrain rice, ≤50% and white rice, >50%	370 (4.3)	158 (5.6)	
	White rice 100%	2,353 (28.8)	744 (28.0)	

Values are presented as number (weighted %).

1 Chi-square test.

2 Using the independent t-test.

**Table 2.** Logistic regression analysis for periodontitis according to the proportion of white rice and multigrain rice consumption^1^

Variables		Model 1 (n=12,450)	Model 2 (n=11,767)	Model 3 (n=11,066)
Ratio of multigrain rice and white rice				
	Multigrain rice 100%	0.76 (0.66, 0.87)^*^	0.78 (0.68, 0.90)^*^	0.80 (0.69, 0.93)^*^
	Multigrain rice, >50% and white rice, ≤50%	0.85 (0.72, 1.01)	0.90 (0.75, 1.07)	0.88 (0.73, 1.06)
	Multigrain rice, ≤50% and white rice, >50%	0.85 (0.67, 1.09)	0.84 (0.65, 1.10)	0.90 (0.69, 1.19)
	White rice 100%	1.00 (reference)	1.00 (reference)	1.00 (reference)
	p for trend	<0.001	0.001	0.004

Values are presented as odds ratio (95% confidence interval).

1 Model 1 was adjusted for age and sex; Model 2 was additionally adjusted for sociodemographic status variables (level of education and household income); Model 3 was additionally adjusted for general health indicators, such as smoking habit, diabetes, hypercholesterolemia, hypertension, and body mass index, and oral health-related variables such as frequency of toothbrushing and use of oral hygiene devices such as floss and interdental brush.

* p<0.05.

**Table 3.** Logistic regression analysis of the effects of consuming rice according to the presence of periodontitis by sex, age and diabetes^1^

Variables		n	Multigrain rice 100%	Multigrain rice, >50% and white rice, ≤50%	Multigrain rice, ≤50% and white rice, >50%	White rice 100%
Sex						
	Male	4,316	0.85 (0.70, 1.04)	0.83 (0.66, 1.05)	0.93 (0.68, 1.28)	1.00 (reference)
	Female	6,750	0.76 (0.62, 0.93)^*^	0.97 (0.72, 1.30)	0.82 (0.51, 1.34)	1.00 (reference)
Age (yr)						
	19-39	4,542	0.79 (0.60, 1.04)	0.76 (0.55, 1.06)	0.70 (0.39, 1.25)	1.00 (reference)
	40-64	6,809	0.84 (0.71, 0.99)^*^	0.89 (0.71, 1.11)	0.91 (0.66, 1.24)	1.00 (reference)
Diabetes mellitus						
	Normal	8,088	0.75 (0.62, 0.91)^*^	0.85 (0.68, 1.07)	0.91 (0.64, 1.27)	1.00 (reference)
	Impaired fasting glucose	2,177	0.80 (0.61, 1.06)	0.83 (0.57, 1.20)	0.97 (0.56, 1.66)	1.00 (reference)
	Diabetes	801	1.52 (0.90, 2.56)	1.58 (0.81, 3.06)	0.96 (0.35, 2.63)	1.00 (reference)

Values are presented as odds ratio (95% confidence interval).

1 Adjusted for age, sex, socio-demographic status variables (level of education and household income), general health indicators (smoking habit, diabetes, hypercholesterolemia, hypertension, and body mass index), and oral health-related variables (frequency of toothbrushing and use of oral hygiene devices such as floss and interdental brush).

* p<0.05.

**Table 4.** Results of the chi-square test for the effects of consuming rice according to sex, age and diabetes

Variables	Sex		Age (yr)		Diabetes mellitus		
	Male	Female	19-39	40-64	Diabetes	Impaired fasting glucose	Normal
Multigrain rice 100%	2,356 (39.2)	5,640 (64.1)	2,550 (43.9)	5,446 (58.1)	589 (60.8)	1,342 (50.1)	4,941 (51.8)
Multigrain rice, >50% and white rice, ≤50%	1,115 (19.2)	901 (10.9)	837 (15.4)	1,179 (14.8)	126 (15.9)	350 (16.3)	1,191 (14.5)
Multigrain rice, ≤50% and white rice, >50%	391 (7.0)	220 (2.2)	211 (3.9)	400 (5.3)	36 (5.0)	109 (5.2)	358 (4.4)
White rice 100%	1,831 (34.6)	1,800 (22.8)	1,939 (36.8)	1,692 (21.8)	136 (18.3)	602 (28.4)	2,222 (29.3)
p-value	<0.001		<0.001		<0.001		

Values are presented as number (%).

**Table 5.** Average blood glucose level according to the type of rice intake stratified by diabetes and periodontitis status

Diabetes mellitus		Fasting blood glucose average according to presence of periodontitis	
		No	Yes
Diabetes			
	Multigrain rice 100%	144±42	147±44
	Multigrain rice, >50% and white rice, ≤50%	157±52	145±39
	Multigrain rice, ≤50% and white rice, >50%	156±49	145±31
	White rice 100%	157±58	164±64
Impaired fasting glucose			
	Multigrain rice 100%	106±6	108±7
	Multigrain rice, >50% and white rice, ≤50%	107±6	108±6
	Multigrain rice, ≤50% and white rice, >50%	107±7	109±8
	White rice 100%	106±6	107±6
Normal			
	Multigrain rice 100%	89±6	90±6
	Multigrain rice, >50% and white rice, ≤50%	89±6	91±6
	Multigrain rice, ≤50% and white rice, >50%	90±6	91±5
	White rice 100%	89±6	90±5

Values are presented as mean±standard deviation.
